# Exposure to bloom-like concentrations of two marine *Synechococcus* cyanobacteria (strains CC9311 and CC9902) differentially alters fish behaviour

**DOI:** 10.1093/conphys/cou020

**Published:** 2014-06-05

**Authors:** T. J. Hamilton, J. Paz-Yepes, R. A. Morrison, B. Palenik, M. Tresguerres

**Affiliations:** 1 Department of Psychology, MacEwan University, Edmonton, Canada; 2 Marine Biology Research Division, Scripps Institution of Oceanography, University of California, San Diego, CA, USA; 3 Institut de Biologie de I'Ecole Normale Supérieure, CNRS, UMR 8197, 46 rue d'Ulm, 75230 Paris, France

**Keywords:** Fish physiology, harmful algal bloom, scototaxis

## Abstract

Coastal California experiences large-scale blooms of *Synechococcus* cyanobacteria, which are predicted to become more prevalent by the end of the 21st century as a result of global climate change. This study investigated whether exposure to bloom-like concentrations of two *Synechococcus* strains, CC9311 and CC9902, alters fish behaviour. Black perch (*Embiotoca jacksoni*) were exposed to *Synechococcus* strain CC9311 or CC9902 (1.5 × 10^6^ cells ml^−1^) or to control seawater in experimental aquaria for 3 days. Fish movement inside a testing arena was then recorded and analysed using video camera-based motion-tracking software. Compared with control fish, fish exposed to CC9311 demonstrated a significant preference for the dark zone of the tank in the light–dark test, which is an indication of increased anxiety. Furthermore, fish exposed to CC9311 also had a statistically significant decrease in velocity and increase in immobility and they meandered more in comparison to control fish. There was a similar trend in velocity, immobility and meandering in fish exposed to CC9902, but there were no significant differences in behaviour or locomotion between this group and control fish. Identical results were obtained with a second batch of fish. Additionally, in this second trial we also investigated whether fish would recover after a 3 day period in seawater without cyanobacteria. Indeed, there were no longer any significant differences in behaviour among treatments, demonstrating that the sp. CC9311-induced alteration of behaviour is reversible. These results demonstrate that blooms of specific marine *Synechococcus* strains can induce differential sublethal effects in fish, namely alterations light–dark preference behaviour and motility.

## Abstract (precis)

Coastal California regularly experiences blooms of *Synechococcus* cyanobacteria. We found that black perch exposed to one strain (CC9311), but not another (CC9902), spent more time in the dark area of a tank, and moved less. Our results demonstrate that blooms of specific strains of marine cyanobacteria can differentially affect fish.

## Introduction

Phytoplanktonic organisms are major primary producers that are essential components of aquatic ecosystems. In certain environmental conditions, including anthropogenic pollution, certain species of phytoplankton can achieve dense populations that may be harmful for other aquatic life due to their production of toxic secondary metabolites and/or generation of hypoxic or anoxic conditions. These phenomena, known as harmful algal blooms, can be caused by many diverse marine and freshwater phytoplanktonic species, including cyanobacteria, dinoflagellates and diatoms (Landsberg, 2002). In some cases, the effects of harmful algal blooms are due to toxins that produce neurological, gastrointestinal or hepatic problems. For example, the dinoflagellate *Karenia brevis*, which is responsible for the typical ‘red tides’, secretes brevetoxin that can bioaccumulate in tissues of consumer species and eventually affect humans (Watkins *et al.*, 2008). Other toxins produced by phytoplanktonic organisms are domoic acid (a neurotoxin produced by some diatoms of the genus *Pseudo-nitzschia*), saxitoxin (a neurotoxin produced by some marine dinoflagellates and freshwater cyanobacterial strains) and microcystins (hepatotoxins produced almost exclusively by some freshwater cyanobacterial strains; Landsberg, 2002; Salierno *et al.*, 2006; Bakke and Horsberg, 2007; O'Neil *et al.*, 2012; Hackett *et al.*, 2013). As toxin-producing phytoplanktonic species can be very abundant in certain marine environments, they have the potential to affect marine life negatively.

Marine *Synechococcus* are unicellular cyanobacteria that are abundant throughout the world's oceans and are significant primary producers in coastal environments, where they occupy an important position at the base of the marine food web (Johnson and Sieburth, 1979; Waterbury *et al.*, 1986; Olson *et al.*, 1990; Zwirglmaier *et al.*, 2008; Flombaum *et al.*, 2013). The *Synechococcus* genus is ecologically and genetically diverse and is composed of multiple ‘clades’ or species (Waterbury *et al.*, 1986; Ferris and Palenik, 1998; Tai and Palenik, 2009; Ahlgren and Rocap, 2012). The four best-understood *Synechococcus* clades, I–IV, are found in distinct environments throughout the global ocean (Scanlan *et al.*, 2009). Clades I and IV are often found together and typically dominate temperate coastal environments, including the coastal Southern California Bight (Brown and Fuhrman, 2005; Zwirglmaier *et al.*, 2008; Tai and Palenik, 2009; Tai *et al.*, 2011). These are the two predominant clades in the *Synechococcus* blooms measured at the Scripps Institution of Oceanography Pier (La Jolla, CA, USA) in late spring, which last ∼1 week and show peak abundances around 6 × 10^5^ cells ml^−1^ (Tai and Palenik, 2009). Clade IV is typically more abundant throughout the year, whereas clade I can become dominant just before the annual spring bloom (Tai and Palenik, 2009; Tai *et al.*, 2011).

Despite the ecological importance of marine *Synechococcus*, very little is known about their toxic effects on other marine organisms. Some marine intertidal *Synechococcus* strains from the Portuguese coast, which do not belong to clades I–IV, produce substances with neurotoxic effects in mice, such as decreased respiratory activity and a general loss of equilibrium (Martins *et al.*, 2005). Some Portuguese coastal *Synechococcus* strains also produce substances with negative effects on invertebrates (Martins *et al.*, 2007); however, the identity of these compounds is unknown. Recently, certain Southern Californian *Synechococcus* strains have been reported to inhibit the growth of other *Synechococcus* strains, very probably due to secreted toxins or secondary metabolites (Leao *et al.*, 2012; Paz-Yepes *et al.*, 2013). Specifically, *Synechococcus* sp. CC9311 from clade I inhibited the growth of sp. WH8102 by unknown secreted molecules. However, it is not known whether this *Synechococcus* strain produces molecules that are also harmful to animals.

The light–dark preference test, also known as the scototaxic test, is a common method to measure anxiety in fish (Maximino *et al.*, 2010) and whether pharmacological compounds alter anxiety (Gerlai *et al.*, 2006; Mathur and Guo, 2011; Maximino *et al.*, 2011). Most of these studies have been performed on freshwater fish; however, this test has recently been used to examine alterations of behaviour in marine fish as well (Hamilton *et al.*, 2014). In that study, exposure to elevated CO_2_/low-pH seawater increased dark preference in juvenile rockfish (*Sebastes diploproa*), a behavioural change that was linked to increased anxiety.

The aim of this study was to determine whether exposure to bloom-like concentrations of two *Synechococcus* strains (sp. CC9311 from clade I and sp. CC9902 from clade IV) alter the behaviour of black perch (*Embiotoca jacksoni*), which are native to coastal California.

## Materials and methods

### Bacterial strains and growth conditions

*Synechococcus* sp. strains CC9311 (clade I) and CC9902 (clade IV) were grown in SN medium (Waterbury and Willey, 1988) made with seawater from the Scripps Pier (Scripps Institution of Oceanography, La Jolla, CA, USA). Cyanobacterial cultures were incubated at 25°C with a constant illumination of 40 μE m^−2^ s^−1^ and were maintained as 1 l cultures in 2.8 l glass flasks with regular stirring. These strains have been kept in culture since their isolation in the 1990s [1993 for CC9311 (Palenik *et al.*, 2006) and 1999 for CC9902 (Dufresne *et al.*, 2008)].

Aliquots of the stocks with a concentration of ∼1 × 10^8^ cells ml^−1^ were added to seawater in aquaria to reach a final concentration of ∼1.5 × 10^6^ cells ml^−1^. The final concentration was estimated from cell counts using a Petroff-Hausser chamber (Hausser Scientific, Horsham, PA, USA) under an epifluorescence microscope. This concentration is comparable to that observed in blooms around the Scripps Pier (Tai and Palenik, 2009) and is the same order of magnitude that has been used in other laboratory studies demonstrating ecologically relevant results (Paz-Yepes *et al.*, 2013). Using a conversion factor of 175 fg carbon cell^−1^ for *Synecochoccus* (Burkhill *et al.*, 1993), the concentration of carbon in each tank due to *Synecochoccus* was 2.6 × 10^4^ mg carbon ml^−1^. The concentration of cyanobacteria in each aquarium was calculated daily and adjusted as needed to maintain similar concentrations among treatment groups, as described above. The control group was exposed to SN culture medium at the same concentration used for the *Synechococcus* groups. Light transmittance, measured in new cultures with the same cyanobacterial density, was identical in the two cultures and <10% lower than the SN control (Supplementary Fig. 1a and b). All cultures look similar to the naked eye (Supplementary Fig. 1c).

### Animal collection and housing

Wild black perch (*Embiotoca jacksoni*) were caught off the coast of San Diego (CA, USA) and housed in groups of 8–12 in three 30 l glass aquaria in the same room as the testing arena described below. Fish average total length was 99.1 ± 6.7 mm (range, 73.5–145.4 mm) and their average weight was 24.0 ± 5.2 g (range, 6.5–68.7 g; *n* = 52). Fish were maintained on a 12 h–12 h light–dark cycle and fed dry fish pellets (New Life Spectrum Small Fish Formula, New Life International Inc., Homestead, FL, USA) *ad libitum* once daily. On experimental trial days, fish were fed after behavioural testing.

To prevent build-up of waste products, depletion of nutrients and major changes in seawater chemistry and to keep *Synechococcus* abundance relatively constant, one-third of the seawater in each tank was replaced with fresh seawater daily. Water temperature (19.1 ± 0.2°C), pH (8.00 ± 0.02) and dissolved oxygen (9.92 ± 0.06 mg l^−1^) in each tank were measured several times daily; none of these variables differed significantly among treatments.

A total of 8–12 fish were randomly assigned to each of three treatment groups: (i) *Synechococcus* sp. CC9311 (clade I); (ii) *Synechococcus* sp. CC9902 (clade IV); and (iii) control. Behavioural testing as described below occurred on day 3 of the exposures.

### Behavioural testing

In order to determine whether the effects of *Synechococcus* strains CC9311 and CC9902 on fish behaviour differed from each other or from the control treatment, we conducted a series of behavioural experiments using the light–dark (scototaxic) test. The light–dark testing arena was created by affixing panels of white and black non-reflective and non-toxic foam board to the sides and bottom of a ∼67 l glass aquarium (74 cm × 30 cm × 30 cm). Ambient seawater was used to fill the arena to a depth of 13 cm, and the seawater was replaced after every 10 testing trials. All testing took place between 09.00 and 18.00 h in a sound-controlled room with moderate overhead lighting. White corrugated cardboard was placed around the arena to minimize shadows in the testing arena and to eliminate external visual stimuli.

At the beginning of each trial, a fish was netted from one of the three treatment tanks and gently placed into the centre of the arena, perpendicular to the long axis to avoid biasing (Holcombe *et al.*, 2013, 2014; Hamilton *et al.*, 2014). Trials began within 5 s of placing the fish into the arena and were 10 min in duration. Fish movement was detected, recorded and analysed using a monochrome CCD camera located ∼1.5 m above the arena and the differencing method in EthoVison XT version 7.0 (Noldus Information Technology, Leesburg, VA, USA). For each fish, we quantified time spent in the light and dark zones (in seconds), time spent in the middle zone (in seconds; 59 cm × 20 cm area in the centre of the arena) as a proxy for thigmotaxis, number of zone transitions, average velocity (in centimetres per second), meandering (in degress per second) and time spent immobile (in seconds; 5% threshold, as defined by Pham *et al.*, 2009).

This exposure protocol was then repeated on a second batch of fish, yielding two experimental sets (ES1 and ES2). In the second experiment, we additionally determined whether the fish could recover from the effects of *Synechococcus* strains CC9311 by placing all groups of fish into normal flowing seawater and repeating behavioural testing 3 days later.

All animal handling and experiments followed approved Animal Care Protocol S10320.

### Statistical analysis

All data sets were tested for normality with the D'Agostino–Pearson omnibus test. All normally distributed data passed Bartlett's test for equal variance. Time spent in the dark zone was analysed with one-sample *t* tests (for normally distributed data) or Wilcoxon signed-rank tests to assess statistically significant differences from 300 s, as commonly used in these types of studies (Maximino *et al.*, 2007; Cripps *et al.*, 2011; Mathur and Guo, 2011; Holcombe *et al.*, 2013; Hamilton *et al.*, 2014). For all other variables, one-way analysis of variance (ANOVA) with Tukey's HSD *post hoc* test (for normally distributed data) or Kruskal–Wallis one-way ANOVA with Dunnett's multiple comparison tests were used, with α = 0.05 and 95% confidence intervals, to assess statistically significant differences among treatment groups. To analyse within-group comparisons, Student's unpaired *t* tests or Mann–Whitney *U *tests were used. Data were analysed with GraphPad Prism 4.0B (GraphPad Software Inc., La Jolla, CA, USA) and are presented as mean values ± SEM. Power analyses were performed for average velocity, meandering and time spent immobile using http://www.statisticalsolutions.net/pss_calc.php.

## Results

### Effects of 3 day *Synechococcus* exposure

None of the variables analysed (time spent in the dark zone or middle zone, number of zone transitions, average velocity, meandering and time spent immobile) were significantly different between the two experimental sets of fish (ES1 and ES2) for any of the three treatments (one-way ANOVAs with Tukey's HSD *post hoc* test or Kruskal–Wallis with Dunnett's multiple comparison tests, *P* > 0.05 for all pairwise comparisons); therefore, data from ES1 and ES2 were pooled for subsequent analyses.

**Figure 1:**
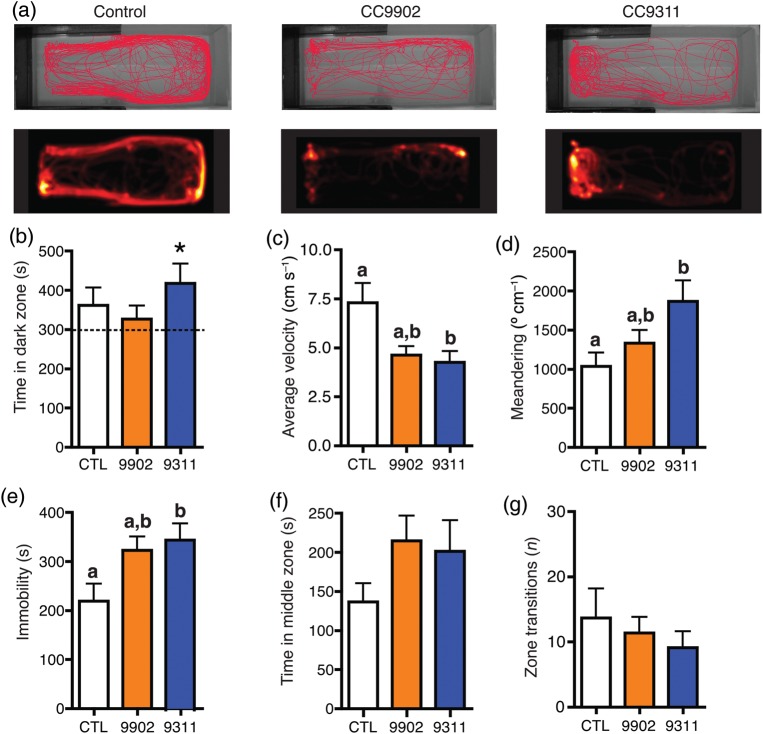
Exposure to *Synechococcus* for 3 days. (**a**) Fish from the control, *Synechococcus* sp. CC9902 and *Synechococcus* sp. CC9311 groups were individually placed in the light–dark preference test arena, and their location was recorded for 900 s. The upper trace illustrates the movement of one representative fish from each treatment over the duration of the trial. The heatmap (below) represents the movement of the same fish throughout the trial and is proportional to the time the fish spent in each pixel. (**b**) Control (CTL) and *Synechococcus* sp. CC9902 (9902) fish did not have a zone preference, whereas *Synechococcus* sp. CC9311 (9311) fish spent significantly more time in the dark zone, indicating increased anxiety. *Statistically significant difference from 300 s, *P* < 0.05. (**c**) Average velocity was significantly lower in the CC9311 group, but not in the CC9902 group, compared with the control group, nor in the CC9311 group compared with the CC9902 group. (**d**) Meandering was significantly greater in the CC9311 group, but not in the CC9902 group, compared with the control group, nor in the CC9311 group compared with the CC9902 group. (**e**) Immobility was significantly higher in the CC9311 group, but not in the CC9902 group, compared with the control group, nor in the CC9311 group compared with the CC9902 group. (**f** and **g**) There were no significant differences among groups in time spent in the middle zone or number of zone transitions. For b–g, control (*n* = 15), CC9902 (*n* = 13) and CC9311 (*n* = 16). Letters indicate statistically significant differences between the treatments (the same letter indicates lack of statistically significant differences).

After 3 days of exposure to treatment conditions, there was no significant preference for the dark zone in either the control group (352 ± 43 s, *P* = 0.1342, *n* = 15) or the *Synechococcus* sp. CC9902 group (327 ± 34 s, *P* = 0.4404, *n* = 13; Fig. 1a and b), but the *Synechococcus* sp. CC9311 group exhibited a significant preference for the dark zone (418 ± 50 s, *P* = 0.0327, *n* = 16; in all cases, one-sample *t* test, difference from 300 s; see Supplementary Videos 1–3).

Average velocity was significantly lower in the *Synechococcus* sp. CC9311 group (4.3 ± 0.6 cm s^−1^, *n* = 16), but not in the *Synechococcus* sp. CC9902 group (4.6 ± 0.5 cm s^−1^, *n* = 13), compared with the control group (7.3 ± 1.0 cm s^−1^, *n* = 15; Kruskal–Wallis test, *H*(2, 41) = 7.379, *P* = 0.0250, followed by Dunnett's multiple comparison *post hoc* test, *P* < 0.05; Fig. 1c). Furthermore, meandering was significantly higher in the *Synechococcus* sp. CC9311 group (1815 ± 281° cm^−1^, *n* = 16), but not in the *Synechococcus* sp. CC9902 group (1332 ± 170° cm^−1^, *n* = 13), compared with the control group (1037 ± 175° cm^−1^, *n* = 15; one-way ANOVA, *F*(2, 41) = 4.014, *P* = 0.0256, followed by Tukey's HSD *post hoc* test, *P* < 0.05; Fig. 1d). Time spent immobile was significantly longer in the *Synechococcus* sp. CC9311 group (349 ± 34 s, *n* = 16), but not in the *Synechococcus* sp. CC9902 group (323 ± 28 s, *n* = 13), compared with the control group (219 ± 36 s, *n* = 15; one-way ANOVA, *F*(2, 41) = 3.356, *P* = 0.0193, followed by Tukey's HSD *post hoc* test, *P* < 0.05; Fig. 1e).

Time spent in the middle zone was not significantly different among the three groups (CC9311, 201 ± 40 s, *n* = 16; CC9902, 215 ± 32 s, *n* = 13; and control, 137 ± 34 s, *n* = 15; Kruskal–Wallis test, *H*(2, 41) = 3.462, *P* > 0.05; Fig. 1f), indicating that there were no differences in thigmotaxis (time spent near the tank walls) among groups. The number of transitions between the light and dark zones was also not significantly different among groups (CC9311, 9 ± 3, *n* = 16; CC9902, 11 ± 3, *n* = 13; and control, 14 ± 5, *n* = 15; Kruskal–Wallis test, *H*(2, 41) = 1.141, *P* > 0.05; Fig. 1g).

Given that the average velocity, meandering and immobility in fish exposed to CC9902 were not significantly different from either the control or the CC9311 group, we performed two-tailed *post hoc* power analyses to calculate the possibility of committing a type II statistical error. These analyses indicated that our statistical tests had a power of 0.76 for average velocity, 1.00 for meandering and 0.77 for immobility. In other words, the chances of not detecting a significant difference due to sample size and variability were 24, 0 and 23%, respectively.

### Recovery in normal seawater

**Figure 2:**
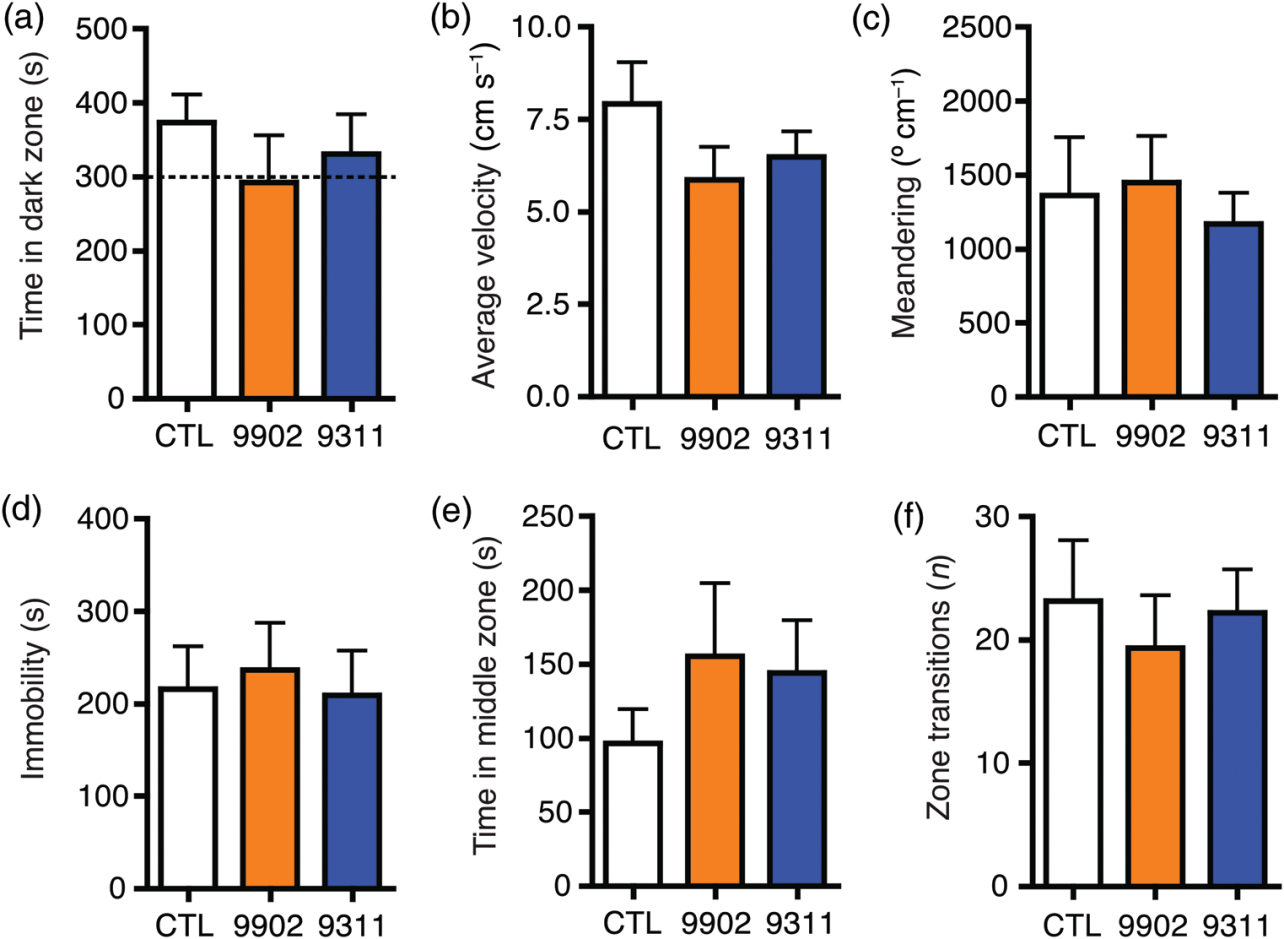
Recovery after *Synechococcus* exposure. After the 3 day exposure to *Synechococcus* sp. CC9902, *Synechococcus* sp. CC9311 or control conditions, all groups were placed back into normal, flowing seawater and retested after 3 days. There were no longer significant differences in time spent in the dark zone (**a**), average velocity (**b**) and meandering (**c**) in the CC9311 group compared with the control group. There were also no significant differences among groups in immobility (**d**), time spent in the middle zone (**e**) and number of zone transitions (**f**).

To investigate whether the effects of *Synechococcus* strains CC9311 and CC9902 were reversible, fish from all groups were placed in normal, flowing seawater and their behaviour was tested again 3 days later. Control fish behaved in an identical manner to the previous trial. In addition, fish that had previously been exposed to *Synechococcus* did not show any significant differences in any of the variables analysed [time spent in the dark zone (one-sample *t*-test, difference from 300 s, *P* > 0.05); average velocity (one-way ANOVA, *F*(2, 22) = 1.296, *P* > 0.05); meandering (one-way ANOVA, *F*(2,22) = 0.2443, *P* > 0.05); time spent immobile (one-way ANOVA, *F*(2, 22) = 0.0907, *P* > 0.05); time spent in the middle zone (Kruskal–Wallis test, *H*(2, 22) = 0.4442, *P* > 0.05); and number of zone transitions (one-way ANOVA, *F*(2, 22) = 0.2257, *P* > 0.05; Fig. 2)].

Finally, we performed a within-group comparison of the effects of *Synechococcus* sp. CC9311 on day 3 of exposure vs. recovery. There were significant differences in the time spent in the dark zone, average velocity, meandering, time spent immobile and number of zone transitions (Student's unpaired *t* test or Mann–Whitney *U* test, *P* < 0.05 for all pairwise comparisons; Table 1). Similar within-group comparisons for both the *Synechococcus* sp. CC9902 group and the control group demonstrated no significant change in any variable (Table 1).

**Table 1:** Comparison of day 3 of exposure with recovery for each group in the second experiment (ES2)

	Control		*Synechococcus* sp. CC9902		*Synechococcus* sp. CC9311	
	Day 3 of exposure (*n* = 8)	Recovery (*n* = 7)	Day 3 of exposure (*n* = 9)	Recovery (*n* = 9)	Day 3 of exposure (*n* = 10)	Recovery (*n* = 9)
Time spent in dark zone (s)	365 ± 70	373 ± 38	344 ± 48	292 ± 64	448 ± 60	331 ± 54*
Mean velocity (cm s^−1^)	6.5 ± 1.6	7.9 ± 1.1	4.4 ± 0.7	5.9 ± 0.9	4.1 ± 0.7	6.5 ± 0.7*
Meandering (° cm^−1^)	1381 ± 261	1363 ± 391	1473 ± 229	1450 ± 312	2110 ± 351	1169 ± 211*
Time spent immobile (s)	293 ± 51	216 ± 46	361 ± 28	237 ± 51	374 ± 40	209 ± 48*
Time spent in middle zone (s)	111 ± 24	97 ± 23	215 ± 43	155 ± 49	135 ± 42	144 ± 35
Number of zone transitions	20.3 ± 7.7	23.1 ± 4.9	14.0 ± 3.7	19.3 ± 4.3	7.2 ± 2.8	22.2 ± 3.5**

*Time spent in the dark zone: statistically significant difference from 300 s, *P* < 0.05. All other parameters: statistically significant differences among treatments (Student's unpaired *t* tests or Mann–Whitney *U* tests, *P* < 0.05). **P < 0.01.

## Discussion

Given that different *Synechococcus* strains may produce different toxins and secondary metabolites (Leao *et al.*, 2012; Paz-Yepes *et al.*, 2013) and that toxins from other planktonic organisms have been shown to induce neurological and behavioural changes in other aquatic organisms (Baganz *et al.*, 2004; Zagatto *et al.*, 2012; Zhang *et al.*, 2013; Ferrâo-Filho *et al.*, 2014), we hypothesized that exposure to two distinct *Synechococcus* strains would differentially alter fish behaviour and locomotion. Our results demonstrated a significant effect of *Synechococcus* strain CC9311, but not CC9902, on dark preference of black perch (*E. jacksoni*). Furthermore, exposure to both *Synechococcus* strains decreased average velocity, whereas it increased meandering and immobility. However, the effects were statistically significantly different from control fish only in the CC9311 group.

There are at least two potential, not mutually exclusive, explanations for the effect of *Synechococcus* sp. CC9311 on the alteration of fish behaviour, as follows: (i) CC9311 may produce a toxin that is capable of entering the circulatory system of the fish and crossing the blood–brain barrier to exert a direct effect on neuronal activity, resulting in decreased velocity and increased dark preference; and (ii) CC9311 may produce a toxin(s) or secondary metabolite(s) that induces some other negative effect (i.e. sickness), and the stress response of the fish is to move into a safer area (dark zone) and to move less in order to promote recovery. In any case, the end result is clear; exposure to *Synechococcus* sp. CC9311 for 3 days induced a change in the decision-making of the fish, altered locomotion and increased anxiety behaviour.

Our results indicate that *Synechococcus* sp. CC9311 has a significant effect on the behaviour of marine fish at a concentration of ∼1.5 × 10^6^ cells ml^−1^ after a 3 day exposure. Although exposure to *Synechococcus* sp. CC9902 for the same amount of time did not induce any significant changes in light–dark preference in comparison to the control group, it did affect some other variables with a similar trend in comparison to CC9311-treated fish. Specifically, average velocity was reduced, while meandering and time spent immobile were increased. Although these variables were not significantly different compared with control fish, they were also not significantly different compared with fish exposed to CC9311 (Fig. 1). Additionally, power analyses indicated that the possibility of type II statistical error for each of these variables was non-existent to relatively low (24% for average velocity, 0% for meandering and 23% for immobility). Thus, while we cannot rule out the possibility that CC9902 mildly affects a subset of the parameters tested, we confidently conclude that CC9311 induces differential effects on black perch in our experimental conditions. This conclusion is further strengthened by the fact that when fish were placed back into clean seawater, the only group to show a significant change in behaviour (‘recovery’) was the CC9311 group, again indicating a lesser effect of CC9902.

There are at least three potential explanations for these results, as follows: (i) the two cyanobacterial strains produce the same toxins, but CC9311 produces these toxins in larger amounts; (ii) CC9311 and CC9902 produce different toxins that induce different effects in fish; and (iii) CC9311 and CC9902 produce some toxins in common, but CC9311 produces additional toxins responsible for the differential effects on fish behaviour (for example, dark preference).

Although the environment can also have profound effects on fish behaviour (Magnhagen, 2008), these effects are typically seen upon light disturbances much greater than in our study (e.g. Long and Rosenqvist, 1998; Järvenpää and Lindström, 2004; Wong *et al.*, 2007). Regardless, the two *Synechococcus* cultures induced differential behavioural effects in our experiments, so at the very least, light cannot explain the effects of CC9311. Thus, we can rule out illumination differences caused by the cyanobacteria as the cause for the altered behaviour. Other factors to consider are O_2_, CO_2_ and pH levels. While we kept those factors constant by vigorous aeration and daily water changes, *Synechococcus* blooms almost certainly will change those parameters in the wild, which could have synergistic effects with the presumed toxin(s) or metabolite(s) on fish behaviour.

The effects of exposure to *Synechococcus* sp. CC9311 on black perch are similar to the locomotor deficits observed in freshwater killifish and zebrafish due to toxins from harmful algal blooms (Salierno *et al.*, 2006; Kist *et al.*, 2011; Zhang *et al.*, 2013) and to the alteration of swimming behaviour in larval herring exposed to saxitoxin (Lefebvre *et al.*, 2005) and in crustaceans exposed to cyanobacterium *Cylindrospermopsis raciborskii* (Ferrâo-Filho *et al.*, 2014). These behavioural changes may be explained by several generic mechanisms. In response to toxins, fish may adjust their metabolism to promote biotransformation of the toxin (Zhang *et al.*, 2013); the general decrease in metabolism would decrease energy supply and have a downstream effect on overall movement. Another explanation might be the direct effect of a neurotoxin. For example, paralytic shellfish poisons contain aphatoxins that decrease the action of acetylcholinesterase in the brain (Zhang *et al.*, 2013); the resulting abnormally high acetylcholine levels would have multiple effects in the central nervous system. Many other neurotoxins present in the marine environment, including saxitoxin, brevetoxin and domoic acid, have also been suggested to alter cognitive functioning by differentially altering metabolic activity in the brain (Bakke and Horsberg, 2007); however, no behavioural tests have been performed to verify the presumed change in cognitive ability. In summary, harmful algal blooms producing toxins are capable of inducing either an activation or a depression of activity throughout the brain (Salierno *et al.*, 2006), which could have widespread neurological implications.

The sublethal effects of environmental toxins, such as alterations of fish behaviour, are not as easily detectable as their lethal effects. However, behavioural changes can have significant effects on the life history of an animal. As shown in this and previous studies (Maximino *et al.*, 2010; Hamilton *et al.*, 2014), the light–dark test is a valuable method for assessing behavioural changes in response to environmental stressors by measuring swimming performance parameters, such as velocity, meandering and immobility. Importantly, this test is also capable of examining location-preference behaviour, which is a proxy for anxiety stress. Therefore, the use of the light–dark test is a valuable tool to detect and quantify sublethal effects caused by diverse stressors in marine fish.

The concentrations of *Synechococcus* spp. used in this study were consistent with concentrations recorded during blooms off the coast of California (Tai and Palenik, 2009; Tai *et al.*, 2011). Predictions based on global climate change scenarios (Flombaum *et al.*, 2013) suggest that *Synechococcus* blooms may reach higher concentration peaks by the end of the 21st century, which would enhance sublethal effects, such as those observed in this study. As part of their osmoregulatory mechanism, marine fish must drink seawater almost constantly in order to absorb water across the intestine, thus counteracting dehydration (Smith, 1930). Therefore, during blooms, marine fish have no option but also to ingest *Synechococcus*, regardless of their feeding behaviour. Additionally, putative water-borne toxins may be absorbed through the gills, eyes and skin.

Future research should investigate whether the results from our laboratory study also apply to the wild, and what the implications of the observed behavioural disturbances are. The changes in light–dark preference and movement patterns induced by *Synechococcus* exposure could theoretically affect virtually every aspect of fish biology and ecology, including but not limited to feeding, dispersal, reproduction and predation vulnerability. In turn, this research could bring about many more questions. Are all fish species equally affected by exposure to *Synechococcus* blooms? Do the sublethal effects observed in fish also occur in other marine organisms? Do fish and other motile organisms move away from the location where the bloom takes place? In any case, an important implication to be taken from our study is that predicting potential effects caused by *Synechococcus* blooms requires determining the abundance of specific *Synechococcus* strains and not only the total *Synechococcus* spp. abundance.

Our results demonstrate that the effects of *Synechococcus* sp. CC9311 exposure on fish behaviour are reversible after a recovery period of 3 days in normal seawater. This implies that the potential impact of CC9311 blooms in nature would be transient. Future research will investigate the exact timing of the recovery, which is likely to take place at some point between a few hours and 3 days.

To our knowledge, this study provides the first evidence that blooms of specific marine *Synechococcus* strains can induce differential sublethal effects in marine fish. In order to elucidate the mechanism behind the toxicity produced by *Synechococcus* sp. CC9311 further, it will be necessary to isolate the specific toxin(s) responsible for the effects observed. In the future, it will be valuable to examine the effect of harmful algal blooms on the behaviour of fish in the wild.

## Supplementary material

Supplementary material is available at *Conservation Physiology* online.

Supplementary Data
