# Supplementary material for: Exposure to bloom-like concentrations of two marine Synechococcus cyanobacteria (strains CC9311 and CC9902) differentially alters fish behaviour
Source: Conserv Physiol. 2014 Jun 5;2(1):cou020. doi: 10.1093/conphys/cou020 (PMC4732467; doi:10.1093/conphys/cou020)
Supplement: Supplementary Data [file supp_2_1_cou020__index.html]

Exposure to bloom-like concentrations of two marine Synechococcus cyanobacteria (strains CC9311 and CC9902) differentially alters fish behaviour — Supplementary Data 

# Exposure to bloom-like concentrations of two marine *Synechococcus* cyanobacteria (strains CC9311 and CC9902) differentially alters fish behaviour

## Supplementary Data

Supplementary Data

**Files in this Data Supplement:**

- Supplementary Figure 1 - pdf file
- Supplementary Data - Docx file
- Supplementary Video 1 - m4v file
- Supplementary Video 2 - m4v file
- Supplementary Video 3 - m4v file
