# Supplementary figures and images for: Exposure to bloom-like concentrations of two marine Synechococcus cyanobacteria (strains CC9311 and CC9902) differentially alters fish behaviour
Source: Conserv Physiol. 2014 Jun 5;2(1):cou020. doi: 10.1093/conphys/cou020 (PMC4732467; doi:10.1093/conphys/cou020)

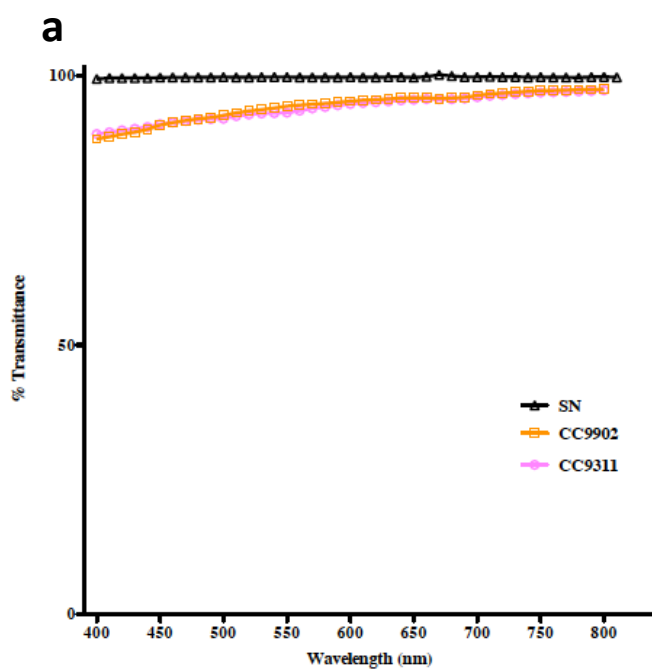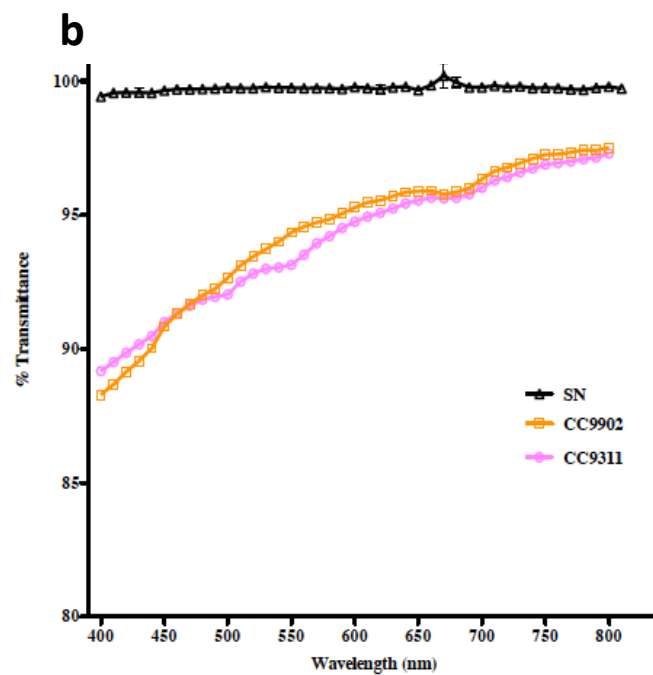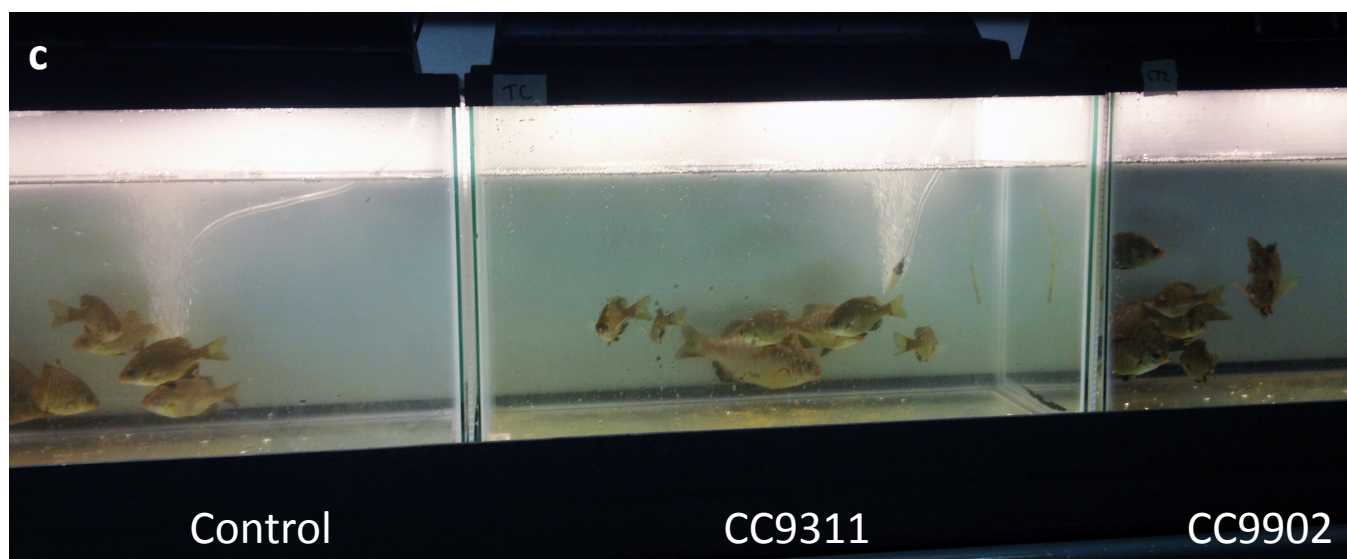

Supplement: Supplementary Data [file supp_cou020_cou020supp_fig1.pdf]
