# Supplementary material for: Exposure to bloom-like concentrations of two marine Synechococcus cyanobacteria (strains CC9311 and CC9902) differentially alters fish behaviour
Source: Conserv Physiol. 2014 Jun 5;2(1):cou020. doi: 10.1093/conphys/cou020 (PMC4732467; doi:10.1093/conphys/cou020)
Supplement: Supplementary Data [file supp_cou020_cou020supp.docx]

**Exposure to a bloom-like concentration of the marine cyanobacterium *Synechococcus* sp. CC9311 (but not CC9902) alters fish behavior**

Hamilton T.J.^1^*^†^, Paz-Yepes J.^2,3†^, Morrison R.A.^2^, Palenik B.^2^, and Tresguerres M.^2^*

**Supplemental Information**

**Supplemental Figure 1.** Effect of cyanobacteria (1.5 10^6^ cells mL^-1^) on seawater turbidity. (a) Light transmittance in the 400-800 nm range, measured every 10 nm. (b) Enlargement of the y-axis (80-100% transmittance). (c) Photograph taken during the experiment showing no visible differences in turbidity between the treatments. CC9902, CC9311=cyanobacterial strains. SN: culture medium alone (control).

**Supplemental Videos 1-3** are representative videos of a 600s trial in the light/dark arena. Fish were individually placed in the center of the arena and released perpendicular to the long axis of the arena. Data recording was started within 5s of release. These videos were taken using a monochrome CCD camera located ~1.5 m above the arena and recorded in EthoVison XT version 7.0 (Noldus, VA, USA). The speed of videos was increased 800% in imovie (Apple, CA). (**a**) Control fish. (**b**) Fish exposed to *Synechococcus* sp. CC9902. (**c**) Fish exposed to *Synechococcus* sp. CC9311.
